# Multiplex Genome Editing in Yeast by CRISPR/Cas9 – A Potent and Agile Tool to Reconstruct Complex Metabolic Pathways

**DOI:** 10.3389/fpls.2021.719148

**Published:** 2021-08-05

**Authors:** Joseph Christian Utomo, Connor Lorne Hodgins, Dae-Kyun Ro

**Affiliations:** Department of Biological Science, University of Calgary, Calgary, AB, Canada

**Keywords:** CRISPR/Cas9, genome editing, multiplex gene integration, *Saccharomyces cerevisiae*, plant specialized metabolites

## Abstract

Numerous important pharmaceuticals and nutraceuticals originate from plant specialized metabolites, most of which are synthesized *via* complex biosynthetic pathways. The elucidation of these pathways is critical for the applicable uses of these compounds. Although the rapid progress of the omics technology has revolutionized the identification of candidate genes involved in these pathways, the functional characterization of these genes remains a major bottleneck. Baker’s yeast (*Saccharomyces cerevisiae*) has been used as a microbial platform for characterizing newly discovered metabolic genes in plant specialized metabolism. Using yeast for the investigation of numerous plant enzymes is a streamlined process because of yeast’s efficient transformation, limited endogenous specialized metabolism, partially sharing its primary metabolism with plants, and its capability of post-translational modification. Despite these advantages, reconstructing complex plant biosynthetic pathways in yeast can be time intensive. Since its discovery, CRISPR/Cas9 has greatly stimulated metabolic engineering in yeast. Yeast is a popular system for genome editing due to its efficient homology-directed repair mechanism, which allows precise integration of heterologous genes into its genome. One practical use of CRISPR/Cas9 in yeast is multiplex genome editing aimed at reconstructing complex metabolic pathways. This system has the capability of integrating multiple genes of interest in a single transformation, simplifying the reconstruction of complex pathways. As plant specialized metabolites usually have complex multigene biosynthetic pathways, the multiplex CRISPR/Cas9 system in yeast is suited well for functional genomics research in plant specialized metabolism. Here, we review the most advanced methods to achieve efficient multiplex CRISPR/Cas9 editing in yeast. We will also discuss how this powerful tool has been applied to benefit the study of plant specialized metabolism.

## Introduction

Plant specialized metabolites (or secondary metabolites) play important roles in enhancing human health and wellness as sources of pharmaceuticals, nutraceuticals, flavors, and fragrances. However, these specialized metabolites and their biosynthetic enzymes are usually available at miniscule levels in plants, making studies of their biosynthesis difficult. The emergence of molecular cloning techniques, next-generation sequencing, omics technology, and synthetic biology in recent decades has accelerated the discovery of specialized metabolic pathways in plants (Siddiqui et al., 2012; Pyne et al., 2019). One of the most powerful tools to study these biosynthetic pathways is the heterologous expression of candidate genes in microorganisms. *Escherichia coli* and *Saccharomyces cerevisiae* are the two major workhorses for microbial pathway reconstruction. While the more fully studied *E. coli* system has been useful to study soluble enzymes, expression of membrane-bound enzymes, such as cytochrome P450s in *E. coli*, is difficult (Leonard and Koffas, 2007). As cytochrome P450s have been discovered to be the major enzyme family driving the chemical diversity of specialized metabolites (Chapple, 1998; Bathe and Tissier, 2019), *S. cerevisiae* with a developed endomembrane system has benefited studies of specialized metabolic pathways, including multiple cytochrome P450s. Several key features of yeast exemplify its practicality (Siddiqui et al., 2012; Pyne et al., 2019). Firstly, efficient yeast transformation techniques simplify the day-to-day uses of yeast. Secondly, yeast has limited endogenous specialized metabolism pathways, which minimizes competition with introduced pathways. Thirdly, yeast partially shares primary metabolism pathways with plants, which means heterologous plant specialized metabolism pathways can be easily plugged into the existing pool of yeast primary metabolite precursors, albeit some flux enhancement may be required. Fourthly, it is relatively safe to work with non-pathogenic yeast, known as one of the generally recognized as safe microbes. Finally, yeast can also carry out some post-translational modifications.

Despite all these advantages, reconstructing complex specialized pathways in yeast is still hindered by two major factors. First, there are a limited number of selection markers for yeast transformation. Selection markers can be recycled, but this is time consuming. Secondly, due to plasmid instability and imbalance, it is often difficult to achieve consistent levels of recombinant proteins in individual cells, which can lead to different degrees of toxicity and metabolic burden in each yeast cell (Da Silva and Srikrishnan, 2012). Thus, the integration of heterologous genes into the yeast genome is preferred for stable expression. Gene integration techniques in yeast, such as *in vivo* homologous recombination and pre-CRISPR endonucleases-based systems (e.g., I-SceI, HO endonuclease, ZFNs, and TALENs), have been extensively developed. These techniques have been reviewed in several excellent articles (Da Silva and Srikrishnan, 2012; David and Siewers, 2015; Yang and Blenner, 2020). However, these integration techniques are relatively laborious as they require selection markers, and there is limited availability of efficient integration sites.

In the past decade, the emergence of endonuclease-based techniques, especially clustered regularly interspaced short palindromic repeats (CRISPR) and its associated protein 9 (Cas9), has revolutionized the field of genome editing. This elegant and simple technique has been applied in various organisms, such as human cells, zebrafish, plants, and *S. cerevisiae* (Doudna and Charpentier, 2014). The simplicity, efficiency, and flexibility of CRISPR/Cas9 have allowed for the expansion of its application to include multiple simultaneous genome-editing events, termed “multiplexing” (Mali et al., 2013). There are some excellent reviews on CRISPR/Cas9 in yeast (Jakočiūnas et al., 2016; Stovicek et al., 2017; Deaner and Alper, 2019; Meng et al., 2020), including some articles focusing on multiplex genome editing (Adiego-Pérez et al., 2019; Malcı et al., 2020). Thus, this review will focus on the most up-to-date advances in multiplex genome editing in *S. cerevisiae* with an emphasis on building the complex pathways of plant specialized metabolites. Specifically, this review focuses on the emergence of CRISPR/Cas9, multiplex gene integration in yeast, current developments in multiplex gene integration using other Cas protein (Cas12a), a brief discussion of other applications of multiplex gene editing, and future perspective of using CRISPR/Cas9 multiplex genome editing for studying plant specialized metabolism.

## CRISPR/Cas9 Development

CRISPR-Cas genome editing is derived from the adaptive immune response of archaea and bacteria and consists of CRISPR genomic sequences and *Cas* genes (Jansen et al., 2002). CRISPR genomic sequences were first discovered in the genome of *E. coli* by Ishino et al. (1987), who found repeating palindromic sequences separated by small, evenly sized, and unique spacer sequences (Ishino et al., 1987). In 2002, CRISPR sequences were shown to be transcribed into CRISPR RNA (crRNA) and the *Cas* genes associated with them were predicted to have nuclease and helicase activity (Jansen et al., 2002; Tang et al., 2002). By 2005, the spacer sequences were determined to be viral sequences (Mojica et al., 2005). In 2007, the CRISPR-Cas adaptive immune response was demonstrated to protect *Streptococcus thermophilus* from invading viruses (Barrangou et al., 2007).

**Figure 1:**
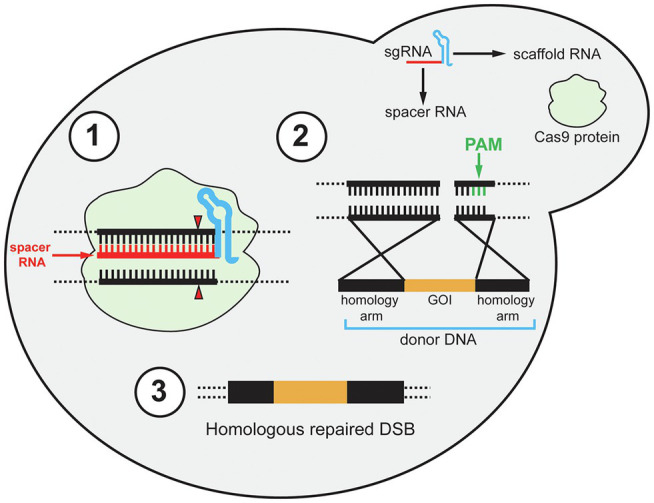
Schematic diagram of the CRISPR/Cas9 mechanism in yeast. The single gRNA (sgRNA) contains two components: scaffold RNA (tracrRNA and structural part of crRNA) and a 20-nt spacer RNA. (1) After the Cas9 protein binds to the sgRNA, Cas9 binds to the target sites in the genomic DNA and undergoes conformational change to cut both strands of the target site 3-bp upstream of the PAM site (red triangle). PAM site is shown in step 2. (2) After a double-stranded break (DSB) is induced, the preferable homology-directed repair (HDR) mechanism repairs the DSB using donor DNA. The donor DNA contains 5' and 3' homology arms and a gene of interest (GOI). The GOI can be non-functional for gene deletion purposes. Note that although the PAM site is emphasized in the second step (green bars), the PAM site is required for Cas9-gRNA complex to recognize the target sites in the first step. (3) After HDR, the DSB is repaired and the GOI is integrated.

Since these initial discoveries, diverse CRISPR-Cas types have been identified but the type II CRISPR system is the most heavily utilized for heterologous gene editing and will be the focus in this review (Makarova et al., 2020). Early studies of the CRISPR immune response of *S. thermophilus* demonstrated that the Cas9 protein uses its catalytic HNH and RuvC-like domains to cleave invading viral DNA (Sapranauskas et al., 2011). At the same time, the requirement of *trans*-activating crRNA (tracrRNA) for the maturation of crRNA in the *Streptococcus pyogenes* CRISPR immune response was demonstrated (Deltcheva et al., 2011). The tracrRNA DNA sequence is located upstream of the CRISPR locus in the bacterial genome. The tracrRNA sequence is complementary to the repeating portion of the crRNA, and when transcribed the tracrRNA and crRNA form a tracrRNA-crRNA duplex. In 2012, the Cas9 protein of *S. pyogenes* (Cas9) was shown to interact with tracrRNA-crRNA duplexes and induce double-stranded breaks (DSBs) in DNA complementary to the spacer sequence, known as the protospacer (Jinek et al., 2012). For recombinant systems, the protospacer sequence represents the target site for gene editing. Also, the tracrRNA-crRNA duplex can be fused in recombinant systems to a single functional transcript, thereby simplifying the heterologous CRISPR-Cas to two components, the single-guide RNA (gRNA) and Cas9 (Figure 1). The gRNA is composed of a 20-nt spacer RNA and scaffold RNA. The scaffold RNA is a fusion of the tracrRNA and the structural repeating portion of the crRNA. A caveat to this system is that a specific sequence called the protospacer adjacent motif (PAM) is required for gRNA recognition of the protospacer (Jinek et al., 2012). The PAM sequence for Cas9 is 5'-NGG-3', while different Cas proteins has different PAM sequences. When binding to a gRNA, Cas9 undergoes a conformational change which forms a channel between the two lobes of the protein (Jinek et al., 2014). This creates room for the binding of the spacer RNA to the target DNA protospacer sequence upstream of the PAM site. After the spacer RNA binds to the target DNA, the HNH domain cleaves the DNA strand complementary to the spacer sequence, and the RuvC-like domain cleaves the opposite strand. The cleavages by HNH and RuvC both occur 3-bp upstream of the PAM creating a blunt DSB (Figure 1; Jinek et al., 2012). The first demonstration of CRISPR/Cas9 genome editing in *S. cerevisiae* followed soon (DiCarlo et al., 2013). Due to highly efficient homology-directed repair (HDR) in *S. cerevisiae*, especially when DSBs are induced, CRISPR/Cas9 is a perfect approach for genomic integrations of foreign genes in yeast (Storici et al., 2003; Shao and Zhao, 2009; Gardner and Jaspersen, 2014). The foreign genes can be transformed as donor DNA, which is a linear DNA fragment that consists of the gene cassette with homology arms at its 5' and 3' ends. The homology arms are DNA sequences that are homologous to the 5' and 3' regions of the DSB sites in the yeast genome (Figure 1). Therefore, by transforming the Cas9 and gRNA expression cassettes, and donor DNA, heterologous gene integration can be achieved.

## CRISPR/Cas9 Multiplex Gene Editing

As soon as CRISPR/Cas9 genome editing was discovered, the idea of simultaneously editing multiple target sites in the genome (multiplex genome editing) was demonstrated in human and mammalian cells (Cong et al., 2013; Mali et al., 2013). In yeast, the first multiplex genome editing was successfully demonstrated soon after the first application of CRISPR/Cas9 genome editing in yeast (Ryan et al., 2014). Thereafter, various methods have been employed to increase the efficiency of multiplex CRISPR genome editing. Here, these methods will be classified based on their gRNA expression systems. In general, there are three common approaches to express gRNA cassettes for multiplex gene editing: (i) expression of multiple gRNAs in a single gRNA cassette with RNA cleaving mechanisms; (ii) expression of multiple gRNAs in multiple gRNA cassettes; and (iii) editing of multiple, pre-defined sequences in the genome by a single gRNA. The studies reviewed here are summarized in Table 1. Unless indicated otherwise, all the plasmids and/or strains are available on request.

**Table 1:** Summary of studies that were reviewed in this manuscript.

Name	^1^Type of editing	Max # edited loci	Maximum # heterologous genes integrated	Total size of genes integrated	Editing efficiency	Genes/pathways integrated	References
CRISPRm (HDV ribozyme)	Deletion (integration)	3 (1)	1	~1.5 kb	20% (85%)	Cellobiose utilization	Ryan et al., 2014
HI-CRISPR (Direct repeats)	Deletion	3	–	100 bp	30–85%	–	Bao et al., 2015
Csy4	Deletion	4	–	120 bp	96%	–	Ferreira et al., 2018
GTR-CRISPR (tRNA)	Deletion	8	–	120 bp	87%	–	Zhang et al., 2019
Mans et al.	Deletion (integration)	6 (2)	6	~15 kb	65% (95%)	Acetyl-CoA biosynthesis	Mans et al., 2015
Modular gRNA	Deletion (integration)	3 (3)	11	~24 kb	64% (4.2%)	Muconic acid biosynthesis	Horwitz et al., 2015
Jakočiūnas et al.	Deletion	4	–	~500 bp	100%	–	Jakočiūnas et al., 2015b
CasEMBLR	Integration	3	3	~18 kb	31%	β-carotene biosynthesis	Jakočiūnas et al., 2015a
CrEdit	Integration	3	3	~18 kb	84%	β-carotene biosynthesis	Ronda et al., 2015
EasyClone-MarkerFree	Integration	3	6	~15 kb	70%	Acetyl-CoA biosynthesis	Jessop-Fabre et al., 2016
Di-CRISPR (delta integration)	Integration	18 (and 10) δ sites	2 (3)	16 kb (24 kb)	95% (85%)	Butadienol biosynthesis and xylose utilization	Shi et al., 2016
CRITGI	Integration	12 Ty sites	1	~5 kb	75%	Pyruvate decarboxylase biosynthesis	Hanasaki and Masumoto, 2019
CMGE	Integration	10 rDNA sites	1	~1.5 kb	46%	GFP expression	Wang et al., 2018
mCAL	Integration	3	3	~4 kb	100%	*HIS3*, *CDC11*, and *SHS1*	Finnigan and Thorner, 2016
Wicket	Integration	3; 6; 9; 12	3	~18 kb	95%; 50%; 50%; 10%	β-carotene biosynthesis	Hou et al., 2018
Landing pads	Integration	4	1	~2 kb	80%	Norcoclaurine biosynthesis	Bourgeois et al., 2018
SGM-CRISPR	Integration	6	6	~15 kb	40%	Kauniolide biosynthesis	Baek et al., 2021
PCR & Go	Integration	5	5	~6 kb	70%	Astaxanthin biosynthesis	Qi et al., 2021
Promiscuous gRNA	Deletion	2	–	120 bp	100%	–	Ferreira et al., 2017

1 Type of editing refers to if the study showed gene deletion (donor DNA is non-functional DNA) or gene integration (donor DNA is functional heterologous DNA).

### Multiple gRNAs in a Single gRNA Cassette With RNA Cleaving Mechanisms

The first strategy of expressing multiple gRNAs for targeting multiple loci is achieved by exploiting RNA cleaving mechanisms from either yeast or other organisms. Using this elegant strategy, multiple gRNAs can be expressed in a single transcript under a single promoter and terminator. Signal sequences are added between each gRNA and can be recognized by RNA cleaving mechanisms thereby producing multiple gRNAs. The multiple gRNAs then bind to multiple target sites simultaneously. Four RNA cleaving mechanisms will be discussed here and included HDV ribozymes, CRISPR direct repeats, Csy4, and tRNA arrays.

#### Hepatitis Delta Virus Ribozyme

**Figure 2:**
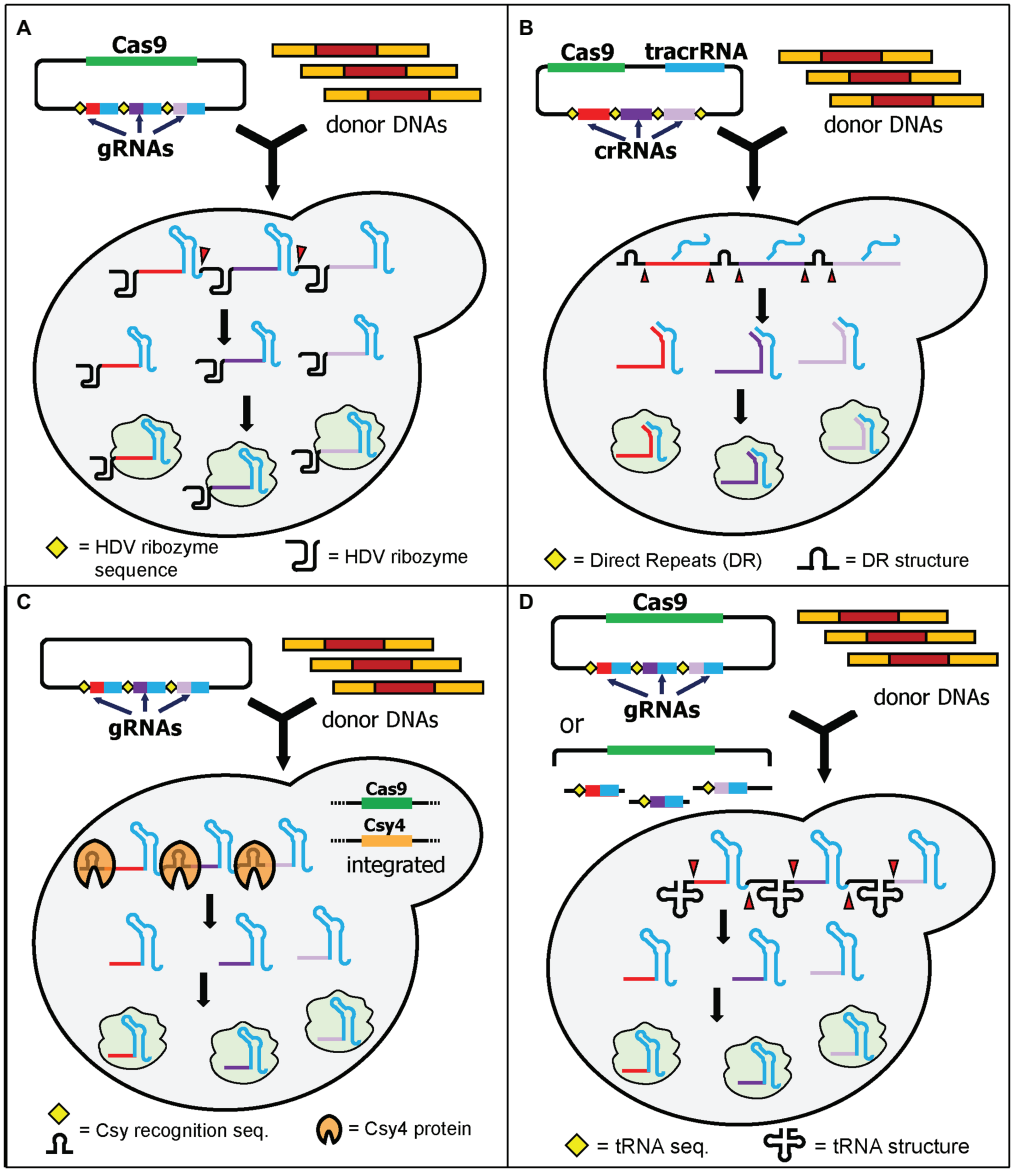
Schematic diagram of multiplex gene editing using multiple gRNAs in a single gRNA cassette with RNA cleaving mechanisms. **(A)** HDV ribozyme self-cleaving mechanism by Ryan et al. After transcription, the HDV ribozyme cuts at its 5' end (red triangles) to produce multiple gRNAs. **(B)** HI-CRISPR by Bao et al. utilized the direct repeats (DR) from *S. pyogenes*. When it is expressed in yeast, the DR will be cleaved (red triangles) by unknown yeast endogenous nucleases and/or RNases, producing multiple gRNAs. Note that the tracrRNA will bind to the structural part of the crRNA before cleaving. **(C)** Csy4 endoribonuclease from *Pseudomonas aeruginosa* can be utilized by providing recognition sites between gRNAs. *Csy4* and *Cas9* were integrated into the yeast genome. Upon transcription, Csy4 recognizes and cleaves the recognition sites. **(D)** Yeast endogenous tRNA can be provided in between the gRNAs. Either fully assembled plasmids or linear plasmids with gRNA fragments can be transformed. The linear plasmid can be assembled *in vivo* using yeast DNA assembly. Upon transcription, the tRNAs are cleaved, resulting in multiple gRNAs (red triangles).

The first demonstration of multiplex genome editing in yeast was demonstrated by Ryan et al. using a plasmid containing a Cas9-expressing cassette and a gRNA cassette containing the self-cleavable hepatitis delta virus (HDV) ribozyme attached to the 5' end of each gRNA (Ryan et al., 2014). The HDV ribozyme cleaves the 5' end of its sequence (Figure 2A). The extra nucleotides from the HDV ribozyme attached to the gRNA do not affect genome-editing efficiency and interestingly the gRNAs modified by the HDV ribozyme significantly improved the efficiency of genome editing. It was shown that the attachment of the HDV ribozyme produces more gRNA transcripts compared to the control system without it (Ryan et al., 2014). This system can knock out a maximum of three loci simultaneously using 120-bp donor DNA with an efficiency around 20 and 80% in diploid and haploid yeast, respectively (Ryan et al., 2014). This approach has been applied to investigate and reconstruct various plant specialized metabolisms, such as tropane alkaloids (Srinivasan and Smolke, 2019, 2020), noscapine (Hafner et al., 2021), and cyanogenic glycosides (Kotopka and Smolke, 2019).

#### Direct Repeats for crRNA Processing

The homology-integrated CRISPR (HI-CRISPR) system separated the expression of tracrRNA and crRNAs rather than combining them in a single gRNA (Bao et al., 2015). The Cas9, tracrRNA, and crRNA (including the 20-nt spacer sequence) cassettes were expressed in one plasmid (available in Addgene). The crRNA cassette contained multiple crRNAs, with each being flanked with direct repeat sequences which mimicked the natural direct repeats of the *S. pyogenes* CRISPR array. After transcription, these repeats were cleaved by the endogenous yeast RNase III and unknown nucleases, resulting in expression of multiple crRNAs from one cassette (Figure 2B). The crRNAs combined with tracrRNAs to form functional gRNAs. This study successfully disrupted up to three different loci in one transformation using 100-bp donor DNA with efficiencies varying from 30 to 85%, depending on the loci targeted.

#### Heterologous Endoribonuclease (Csy4)

A couple of years before utilizing CRISPR/Cas9 for genome editing, the Doudna lab had characterized an endoribonuclease that cleaves direct repeats from pre-crRNA to produce mature crRNAs in *Pseudomonas aeruginosa* (Haurwitz et al., 2010). This endoribonuclease is called Csy4 and has been tested for its functionality to process multiple crRNAs at the same time in various organisms (Qi et al., 2012). The application of Csy4 processing for multiplex genome editing in yeast was first demonstrated by Ferreira et al. (2018). The authors first integrated both Cas9 and Csy4 expression cassettes into the yeast genome. They also built a plasmid containing one gRNA cassette with 28-nt Csy4 recognition sites between each gRNA (Figure 2C). Unlike HDV cleavage, but like the HI-CRISPR system, the Csy4 recognition sites are abolished after cleavage. This results in multiple gRNAs without any additional RNA structures. This approach successfully demonstrated quadruple gene disruptions using 120-bp donor DNA with 96% efficiency. The study also showed that utilization of Csy4 recognition sites in the absence of Csy4 still resulted in 50% efficiency for double-gene deletions (Ferreira et al., 2018). This may suggest that Cas9 might have some flexibility to recognize at least the first two gRNAs in a single long transcript and correctly cleave both genomic DNA targets, albeit with a lower efficiency (Ferreira et al., 2018). The study from Ryan et al. also supports this suggestion as Cas9 can still recognize gRNAs that have additional HDV structures on their 5' ends (Ryan et al., 2014). Alternatively, the endogenous yeast RNase III and nucleases may cleave the Csy4 direct repeats in a similar way to how *S. pyogenes* direct repeats are cleaved in the HI-CRISPR system (Bao et al., 2015). In another independent study, Csy4 processing capability was also demonstrated to express 12 gRNAs simultaneously for CRISPR interference (CRISPRi; McCarty et al., 2019).

#### Yeast Endogenous tRNA Array

Utilizing the endogenous tRNA-processing mechanism for single transcript expression of multiple gRNAs was first demonstrated in rice (Xie et al., 2015). Zhang et al. showed that the tRNA array can also be successfully used to express multiple gRNAs in yeast (Zhang et al., 2019). The system is called GTR-CRISPR (gRNA-tRNA array for CRISPR/Cas9). GTR-CRISPR used a plasmid that contained a Cas9 expression cassette and a gRNA cassette with tRNA_Gly_ sequences between each gRNA. The tRNA sequences were then cleaved during endogenous yeast tRNA processing, resulting in the release of multiple gRNAs from a single transcript (Figure 2D). The authors tested two different arrays to disrupt eight genes at the same time. One array used a single promoter to express all gRNAs, while the other array used two promoters to express eight gRNAs (four gRNAs each). The latter approach resulted in an incredible octuple gene deletion with 87% efficiency, compared to 35.5% efficiency for the former arrangement (Zhang et al., 2019). This tRNA array system has been utilized to integrate the pyruvate dehydrogenase complex (PDH) into the yeast genome (Zhang et al., 2020).

Although these RNA cleavage mechanism approaches are valuable, they have a major limitation. The efficiency of multiplex genome editing using this approach is determined by the lowest expression and cleavage efficiency of its gRNAs. One common issue observed from these studies was that the more downstream a gRNA or crRNA is in the transcript, the less efficient it will be (Ryan et al., 2014; Bao et al., 2015; Ferreira et al., 2018; Zhang et al., 2019). As multiplex gene integration requires multiple factors to be efficient (discussed below), the compounding effects of the inefficiencies of RNA cleavage mechanisms can significantly restrict the application of these approaches for multiplex gene integration. Therefore, the application of these approaches is more widespread for multiple gene disruption (e.g., for disrupting endogenous competing pathways) rather than multiple gene integrations for pathway building.

### Expression of Multi-Cassettes gRNAs

The second strategy of expressing multiple gRNAs simultaneously is to express each gRNA with its own cassette. This approach has been simplified by the development of molecular cloning techniques, such as Golden Gate, Gibson Assembly, USER, and *in vivo* DNA assembler (Deaner and Alper, 2019). Readers can refer to a work by Chao et al., who reviewed the current development of molecular cloning and DNA assembly techniques (Chao et al., 2015). These techniques allow the creation of a large plasmid with relatively quick and straightforward steps. The examples that will be discussed below use multiple gRNA cassettes in one plasmid, while Cas9 is expressed separately either by integration into the genome or using a separate plasmid. Thus, these examples will be categorized into two groups: (i) integrated Cas9 expression and (ii) plasmid-based Cas9 expression.

#### Integrated Cas9 Expression

**Figure 3:**
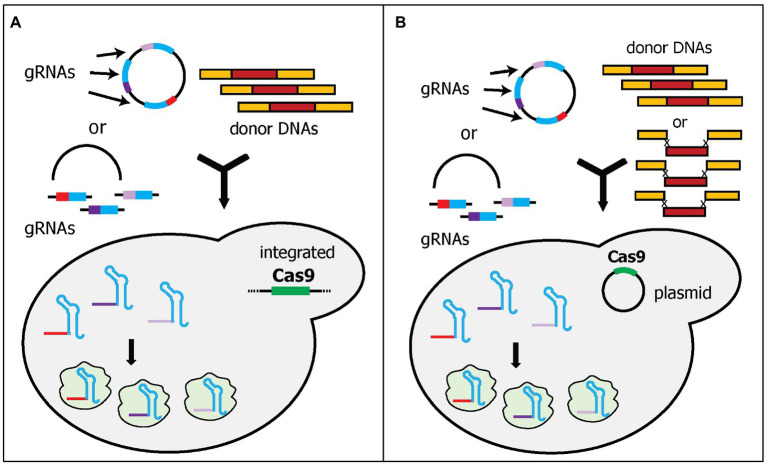
Schematic diagram of multiplex gene editing using multi-cassette gRNA expression. Each gRNA is expressed in one cassette. The gRNA plasmid can be pre-assembled or transformed as linear fragments. The linear fragments can be assembled *in vivo* by yeast endogenous homologous recombination. **(A)** *Cas9* was integrated into the genome to reduce the amount of DNA to be transformed during transformation. **(B)** Cas9 plasmid was pre-transformed into the yeast cells. The studies also demonstrated that donor DNAs can be transformed as separate parts rather than pre-assembled donor DNAs.

In the first category, a Cas9 expression cassette is integrated into the yeast genome, while gRNA cassette(s) are expressed in a plasmid (Figure 3A). The integration of the Cas9 cassette into the genome has some benefits, such as more stable Cas9 expression, maximizing the availability of selection markers for gRNA-expressing plasmids, and reducing the size of the plasmid, thereby, increasing the transformation efficiency (Stovicek et al., 2017). However, the Cas9 cassette in the genome needs to be removed after genome editing. Mans et al. developed a comprehensive toolbox to simplify CRISPR/Cas9 gene editing in yeast (Mans et al., 2015). This toolbox includes (i) the Yeastriction Web tool,^1^ which can help with gRNA design to minimize off-target edits and maximize efficiency; (ii) a set of gRNA expressing plasmids, which includes eight single-gRNA cassette plasmids and eight double-gRNA cassette plasmids with eight different selectable markers (*URA3*, *amdSYM*, *hphNT1*, *kanMX*, *LEU2*, *natNT2*, *HIS3*, and *TRP1*); and (iii) a collection of various haploid, diploid, and auxotrophic CEN.PK strains, into which the Cas9 cassette, under the *TEF1* promoter, has been integrated. Both plasmids and yeast strains were deposited at Euroscarf. The authors showed that using three plasmids, each of which contained two gRNA cassettes, could simultaneously delete six genes with 65% efficiency. Furthermore, they deleted two acetyl-CoA synthetase genes, *ACS1*, and *ACS2*, which are important for cytosolic acetyl-CoA synthesis in yeast. Without these genes, the yeast is not viable unless other sources of acetyl-CoA are supplied. Therefore, they also simultaneously integrated six genes that are part of the *E. faecalis* pyruvate dehydrogenase complex as donor DNAs, to provide the yeast with an alternative acetyl-CoA biosynthesis pathway. All six genes were designed to be assembled *in vivo* into the *ACS2* locus, while the *ACS1* locus was deleted by providing only a 120-bp non-functional donor DNA. Although the efficiency cannot be calculated because failed integrations will not appear as colonies, the study successfully deleted two genes and introduced six heterologous genes (~15 kb) into one locus of the yeast genome in one transformation (Mans et al., 2015).

Similarly, Horwitz et al. also integrated a Cas9 cassette into the yeast genome (Horwitz et al., 2015). They expressed Cas9 under the medium-strength promoter, *FBA1*, as opposed to the high-strength promoter, *TEF1*. More importantly, they used different gRNA plasmid delivery methods. Instead of constructing various plasmids with either one or two gRNAs, Horwitz et al. transformed the yeast cells with one linear plasmid and one, two, or three linear gRNA cassette(s), and then relied upon *in vivo* DNA assembly to circularize the plasmid for gRNA expression (Figure 3A). Interestingly, while Mans et al. attempted this approach and had a very low efficiency, Horwitz et al. showed that triple gene deletions can be achieved with 64% efficiency (Horwitz et al., 2015; Mans et al., 2015). The differences between these two studies are the length of the flanking homology sequences for *in vivo* assembly. Horwitz et al. used 500-bp flanking homology sequences (Horwitz et al., 2015), whereas Mans et al. used only 50-bp flanking homology sequences (Mans et al., 2015). Ultimately, Horwitz et al. also demonstrated the capability of this system to integrate 11 genes (~24-kb) in the muconic acid biosynthetic pathway into three loci of the competing pathway. Although the efficiency was low (4.2%), it shows the ability to delete competing pathways and integrate heterologous pathways simultaneously (Walter et al., 2016).

#### Plasmid-Based Cas9 Expression

Instead of integrating the Cas9 cassette into the yeast genome, the Cas9 cassette and gRNA cassette(s) can be expressed from two different plasmids (Figure 3B). This approach has a major benefit over the integration of Cas9 because the removal of a Cas9 expression plasmid is more straightforward, e.g., using counter selection method. However, the transformation efficiency may decrease due to the large size of DNA required to be delivered if all plasmids are transformed at the same time. Therefore, some of the examples below pre-transformed the Cas9-expressing plasmid before the transformation of the gRNA-expressing plasmid. A collaboration between the groups in the Novo Nordisk Foundation for Biosustainability developed four analogous studies using this system with the main differences being the cloning strategies of gRNA plasmids and donor DNAs (Jakočiūnas et al., 2015a,b; Ronda et al., 2015; Jessop-Fabre et al., 2016). In all four studies, the Cas9 expression cassette was expressed in one plasmid and was pre-transformed into a yeast strain. The constructed yeast strain was then transformed with the gRNA cassette(s)-containing plasmid.

Jakočiūnas et al. built the foundation of their system by systematically selecting target sites using the CRISPy Web tool,^2^ verifying off-target effects, and utilizing USER cloning to assemble the gRNA cassette(s) plasmid (Jakočiūnas et al., 2015b). Using this approach, they successfully generated a collection of 31 mutant strains with one to five endogenous genes being disrupted. These five genes were chosen as single deletions of these genes resulted in a higher metabolite flux toward the mevalonate (MVA) pathway. The screening of the 31 mutant strains resulted in a yeast strain with a titer of more than 10 μM MVA (Jakočiūnas et al., 2015b). The following study combined this system and yeast *in vivo* DNA assembly to bypass the requirements for donor DNA cloning (Jakočiūnas et al., 2015a). In this approach, called CasEMBLR, up to three donor DNAs, containing five parts each (two flanking homology sequences, promoter, gene, and terminator), were transformed with the gRNA-expressing plasmid into yeast cells. The study demonstrated simultaneous integration of three genes in the β-carotene biosynthetic pathway (~18 kb) into the yeast genome with 31% efficiency (Jakočiūnas et al., 2015b).

The last two studies utilized EasyClone for their target sites. EasyClone consists of a set of standardized plasmids that combines USER cloning and Cre-LoxP-based marker recycling system to enable iterative integration of heterologous genes (Jensen et al., 2014). This method uses previously characterized target sites in the genome with high efficiency, low effects on cell growth, and high expression (Jensen et al., 2014). The following studies used and modified EasyClone target sites and plasmids for CRISPR/Cas9 genome editing (Ronda et al., 2015; Jessop-Fabre et al., 2016). In both studies, the Cas9-expressing plasmid was also pre-transformed into yeast cells. Ronda et al. used three characterized target sites from EasyClone to integrate three genes (~18 kb) for β-carotene biosynthesis into the yeast genome with 85% efficiency (Ronda et al., 2015). In the next study, Jessop-Fabre et al. evaluated 11 previously characterized EasyClone target sites for their efficiencies in CRISPR/Cas9 editing (Jessop-Fabre et al., 2016). All plasmids for EasyClone-based CRISPR/Cas9 editing are available at Addgene. They found that the target sites had a 95–100% targeting efficiency. The practicality of this system for multiplex CRISPR/Cas9 editing was successfully demonstrated by integrating either three (one gene in each locus) or six (two genes in each locus) genes in three different acetyl-CoA synthesis pathways from different species into the yeast genome with 60–70% efficiency. Interestingly, targeting different loci in the same chromosome decreases the editing efficiency. By comparing these three different pathways, 3-hydroxypropionic acid production in yeast was optimized (Jessop-Fabre et al., 2016).

Expression of multiple gRNAs in multiple gRNA cassettes for multiplex genome editing has one major advantage compared to the RNA cleaving approach for producing multiple gRNAs from one cassette. This benefit is that the expression of each gRNA is more comparable, which re-directs the rate-limiting steps toward other factors (e.g., yeast transformation efficiency or overall gRNA expression) instead of the lowest gRNA expression. Consequently, more studies utilize this approach for multiplex heterologous gene integrations (Bond and Tang, 2019; Yee et al., 2019). However, one key limitation persists that is the finite amount of gRNA that can be expressed. This is because the more gRNAs expressed by a plasmid, the larger the plasmid will be, which decreases the transformation efficiency. Additionally, multiple gRNAs might still compete for the yeast endogenous RNA transcription machinery and limit the expression of gRNAs transcripts.

### Editing of Multiple Pre-defined Sequences in the Genome by a Single gRNA

So far, the maximum loci that have been demonstrated for simultaneous integration using multiple gRNAs are three (Horwitz et al., 2015). To improve the number of loci for deletions and possibly to increase the number of genes for integrations, other approaches have been developed. In the following approaches, pre-defined target sequences were identified and/or synthetically integrated in the yeast genome. The sequences, which can be endogenous or synthetic, must be found or pre-integrated in multiple sites in the yeast genome. Since multiple loci can be targeted using a single gRNA, it greatly simplifies the integration of the heterologous genes. Several studies have successfully used this approach to integrate multiple genes in multiple loci. These studies can be divided into two categories using either endogenous target sequences or synthetic target sequences.

#### Yeast Endogenous Target Sequences

**Figure 4:**
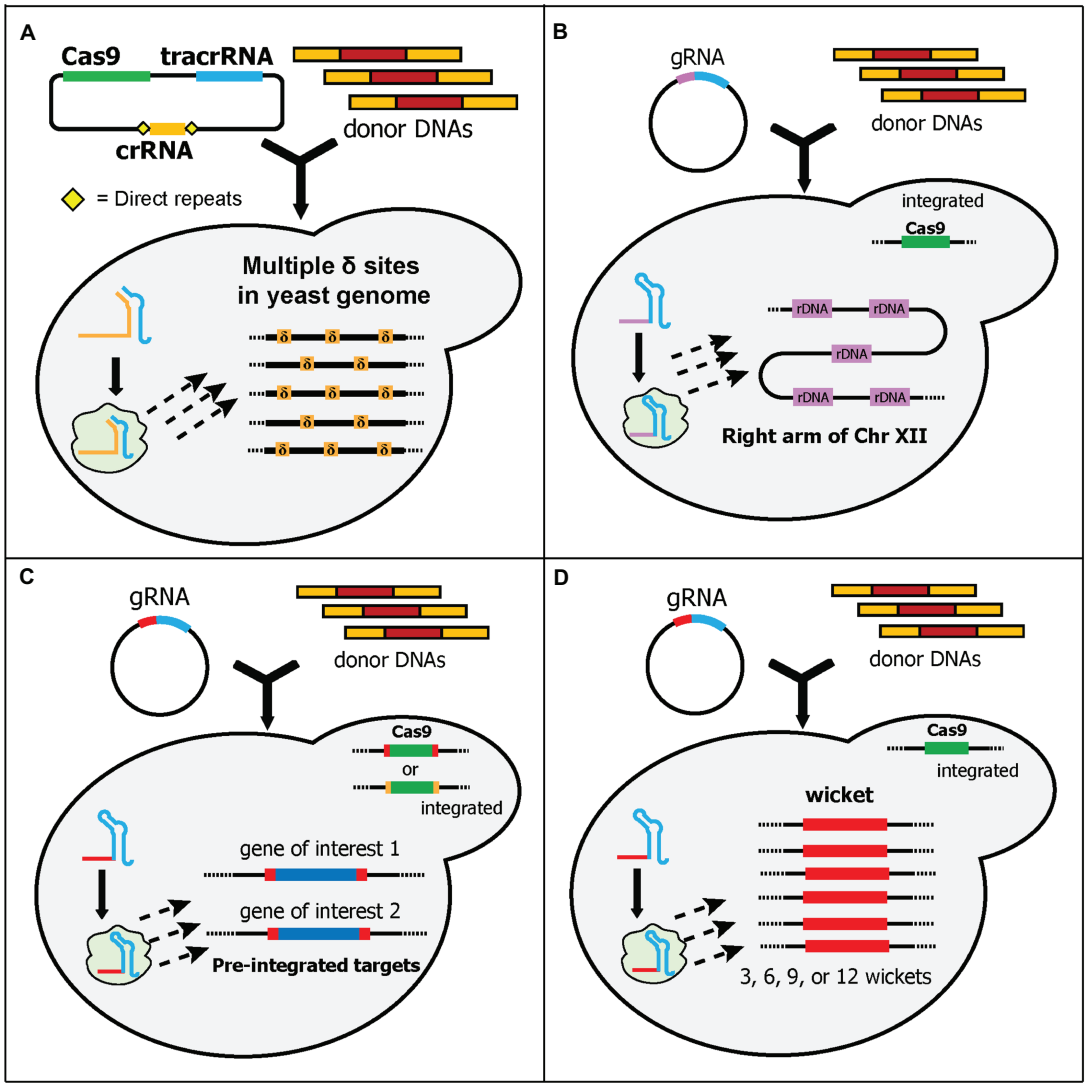
Schematic diagram of multiplex gene-editing pre-defined sequences in the genome using a single gRNA (part 1). **(A)** Shi et al. used δ sites of Ty elements in the yeast genome as the target. More than 30 δ sites (δ, orange boxes) are scattered across all chromosomes in the yeast genome. Therefore, multiple copies of donor DNA can be integrated. Note that they used a separate crRNA and tracrRNA plasmid. **(B)** Wang et al. targeted ribosomal DNA (rDNA, purple boxes) for integration sites. rDNA is located at the right arm of chromosome XII. Around 100–200 copies of rDNA can be found in the yeast genome. *Cas9* was integrated into the genome. **(C)** Finnigan and Thorner generated synthetic target sequences (red or orange boxes) that flanked the genes-of-interest (GOIs) and *Cas9*, which was integrated into the yeast genome. The gRNA cleaves the sequences and causes DSBs at the 5' and 3' ends of GOIs and/or *Cas9*, removing them from the genome. Concurrently, the donor DNAs can be integrated at those sites. Note that although different gRNA sequences can be used, only one gRNA sequence (red box) is shown. **(D)** Hou et al. generated “wicket” sequences (red boxes) which contains a 23-bp synthetic target flanked by 5' and 3' 50-bp synthetic homology arms. Wickets and *Cas9* were integrated into the yeast genome. Yeast strains with 3, 6, 9, or 12 wickets were created. Therefore, multi-copies of donor DNA can be integrated in one transformation.

Some endogenous sequences can be found in multiple sites in the yeast genome. One such endogenous sequence is the delta (δ) site of Ty (transposons of yeast) elements. Like other retrotransposons, Ty can replicate and insert itself in other sites of the yeast genome (Krastanova et al., 2005). Ty is composed of two genes, which are flanked by identical sequences called long terminal repeats (LTRs). There is at least five known Tys (Ty1 to Ty5), and they are scattered throughout the genome. The δ sites refer to the LTRs of Ty1 and Ty2. There are approximately 40 copies of Ty1 and Ty2 LTRs in haploid yeast (Krastanova et al., 2005). Ty1 and Ty2 LTR sequences have been utilized since the 1990s for the integration of multiple gene copies in yeast (Da Silva and Srikrishnan, 2012; Malcı et al., 2020). However, this approach shows poor efficiencies, especially for integrating larger genes (Shi et al., 2016). Inspired by conventional δ site integration, Shi et al. designed a CRISPR/Cas9 system to target these δ sites, called delta integration-based CRISPR (Di-CRISPR; Shi et al., 2016). It exploits HI-CRISPR (Bao et al., 2015) plasmids to separately express crRNA, tracrRNA, and Cas9, although only one crRNA is expressed in Di-CRISPR. The crRNA was designed to target a characterized δ site sequence (Figure 4A). Using this system, the authors demonstrated an astounding 24-kb cassette integration into the δ sites with up to 18 copies of each gene being found in the genome. The 24-kb donor DNA cassette contained seven genes: three xylose utilization genes, three (*R*,*R*)-2,3-butadienol (BDO) biosynthetic genes, and a GFP reporter gene. The resulting strain can utilize xylose as its sole carbon source and produce BDO (Shi et al., 2016). The same group has extended the application of Di-CRISPR for developing automated system to study genotype–phenotype mapping and industrial traits optimization (Si et al., 2017). Recently, another study adopted a similar approach and successfully integrated 25 copies of the BDO biosynthetic pathway and a GFP cassette into δ sites, albeit with a much shorter donor DNA of 4-kb (Huang and Geng, 2020). In another study, Ty elements, instead of δ sites, were targeted for multicopy multiplex genome integration (Hanasaki and Masumoto, 2019). This system, called CRISPR/transposon gene integration, used two plasmids, a Cas9-gRNA expressing plasmid and a donor DNA plasmid. Interestingly, the donor DNA plasmid contains a Ty1 genome sequence, which will be cleaved together with the Ty1 sequences in the genome, by Cas9-gRNA complex. This causes the donor DNA plasmid to be linearized *in vivo* and integrated into the Ty1 sequences. Using this approach, the authors demonstrated the integration of 12 copies of the donor DNA (~5-kb). Additionally, the expression of the genes in the donor DNA can be tuned by exploiting the existence of amino acid markers in the donor DNA plasmid (Hanasaki and Masumoto, 2019).

Another endogenous sequence, ribosomal DNA (rDNA), has also been utilized as a target for multicopy gene integration (Wang et al., 2018). rDNA is located at the *RDN1* locus. The *RDN1* locus is a 1–2 Mb section in chromosome XII of yeast and contains 100–200 copies of a 9.1 kb repeat. Each repeat has regions that encode rRNAs and non-coding regions (Venema and Tollervey, 1999). Wang et al. targeted one of the non-coding regions called the non-transcribed spacers 1 (NTS1) for their system (Wang et al., 2018). In this system, the Cas9 expression cassette was pre-integrated into the genome before the transformation of gRNA plasmid and donor DNA fragment (Figure 4B). Although it has been shown that cleaving multiple targets in the same chromosome may disrupt genome stability and reduce the efficiency of CRISPR/Cas9 (Jessop-Fabre et al., 2016), the authors demonstrated the successful integration of up to 10 copies of a GFP donor DNA cassette with 45% efficiency, and the resulting strain maintained stable copy numbers after 55 generations (Wang et al., 2018). In one study, the CRISPR-based sequential integration of four genes into both δ sites and rDNA successfully increased the production of isobutanol in yeast (Park and Hahn, 2019).

#### Synthetic Target Sequences

Designing an efficient gRNA is one of the most essential factors for successful CRISPR/Cas9 gene editing (DiCarlo et al., 2013). Therefore, the laborious step of testing multiple gRNAs for efficiently integrating heterologous genes in each locus is required. This difficulty is compounded when targeting multiple loci. Moreover, targeting endogenous yeast sequences may have some unintended off-target effects, especially if the targets are not yet tested (Apel et al., 2017). The following studies have developed yeast strains to alleviate these problems by integrating artificial/synthetic gRNA target sequences, which are designed to avoid any potential off-target DSBs, at characterized loci with significant gene expression. The idea of introducing synthetic sequences for a unique target site was first demonstrated by Lee et al. for investigating the effect of a linearized plasmid on integration efficiency (Lee et al., 2015). They termed this synthetic sequence a “landing pad.” Although this study did not attempt to develop a multiplex genome-editing system, the term and concept of a “landing pad” have been used in the following five studies.

In the first study, Finnigan and Thorner generated unique 23 bp synthetic sequences, each of which contained a 20-nt target sequence and 3-bp PAM sequence (Finnigan and Thorner, 2016). Each sequence was integrated into two locations that flanked a gene of interest in the genome (Figure 4C). Thus, expression of the gRNA and Cas9 cuts both synthetic sequences and can be used to substitute the gene of interest with donor DNA fragments. Additionally, they integrated the Cas9 expression cassette, which is also flanked by the synthetic sequence, into the genome. Depending on the design of the synthetic sequences, the editing could result in different final yeast strains (Figure 4C). If the synthetic sequences for the Cas9 cassette and the genes of interest are identical, the final strain will not only contain the substituted genes but also remove the Cas9 cassette in the genome. Otherwise, the final strain could contain the substituted genes but still retain the Cas9 cassette in the genome. Using this system, the authors demonstrated the successful substitution of three genes, with and without the removal of the Cas9 cassette (Finnigan and Thorner, 2016).

The next study from Hou et al. expanded the landing pad concept by synthesizing and integrating an artificial sequence containing a 50-bp 5' homology arm, 20-nt target sequence, 3-bp PAM sequence, and 50-bp 3' homology arm, which they called a “wicket” (Hou et al., 2018). Wicket is a wooden structure in cricket, composed of three stumps (i.e., signifying a left homology arm, central gRNA target sequences, and a right homology arm). Therefore, only one gRNA cassette and one set of universal homology sequence for all donor DNAs are required for editing multiple loci. They also pre-integrated the Cas9 cassette into the genome and constructed yeast strains containing 3, 6, 9, or 12 wickets in various intergenic regions (Figure 4D). To evaluate this system, they used three genes in the β-carotene biosynthetic pathway as donor DNAs. In the process called “pre-assembled integration,” they used one donor DNA containing the 5' and 3' homology arms with all three genes cassettes in the middle. Using this process, the efficiency was between 50 and 100% for strains with 3, 6, and 9 wickets, while the efficiency for 12 wickets was very low. Alternatively, in the process called “cocktail integration,” they used three donor DNAs containing the same 5' and 3' homology arms with only one gene cassette in the middle of each donor DNA. Cocktail integration was intended to control the copy numbers of each donor DNA cassette in the genome. Using this approach, a variety of strains with different amounts of β-carotene were generated, although the efficiency was much lower compared to the pre-assembled donor DNA. Interestingly, they found some tandem duplication events between the donor DNA cassettes in both processes, which caused some strains with three wickets to have up to 20 copies of the donor DNA cassette (Hou et al., 2018).

**Figure 5:**
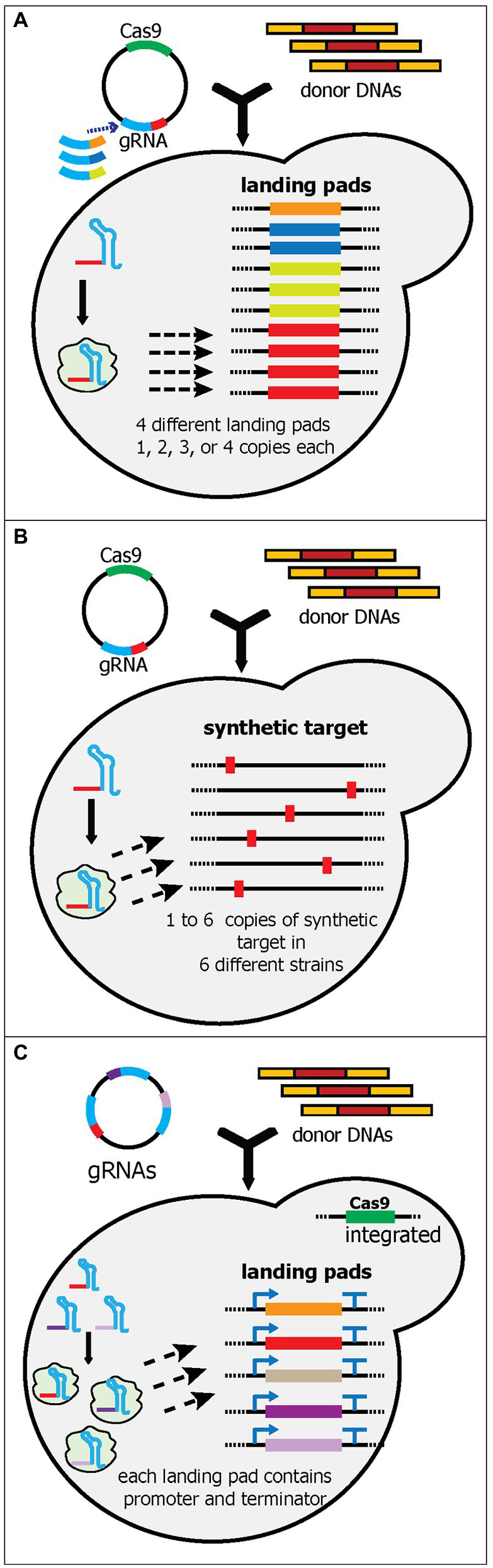
Schematic diagram of multiplex gene-editing pre-defined sequences in the genome using a single gRNA (part 2). **(A)** Bourgeois et al. tested and selected various landing pads sequences. Like wickets, the landing pads contain a 23-bp synthetic target flanked by 280-bp 5' and 3' synthetic homology arms. Four unique landing pads (orange, dark blue, light green, and red boxes) were integrated into 10 characterized loci in the yeast genome. Each landing pad had a different copy number in the genome; i.e., landing pad 1 has one, and landing pad 2 has two. Note that four different plasmids were created, each of which targeted a different landing pad. Only one landing pad gRNA (red) was shown in the yeast cell. **(B)** Baek et al. generated and integrated a 23-bp synthetic sequence (red boxes) into one to six characterized loci in the yeast genome. Six yeast strains were constructed, each of which had from one to six synthetic sequence(s). Therefore, up to six genes could be integrated simultaneously. **(C)** Qi et al. created a system called PCR & Go. Up to eight 23-bp synthetic sequences flanked with unique sets of promoters and terminators were integrated into the yeast genome. The gRNA plasmid contains multiple gRNA cassettes instead of a single gRNA. However, donor DNA preparation can be simplified as unique sets of promoters and terminators had already been integrated into the genome.

The study by Bourgeois et al. attempted to build a yeast strain that can be used to compare and optimize the copy number of heterologous gene integration. To achieve this, they meticulously evaluated 10 synthetic landing pads (with 280-bp 5' and 3' homology arms and a 23-nt synthetic target sequence) and 16 genomic loci for their integration efficiency and gene expression levels (Bourgeois et al., 2018). After the rigorous characterization, they ranked and picked four synthetic landing pads and 10 genomic loci to be used. The four landing pads were integrated into one, two, three, and four genomic loci, respectively (Figure 5A). As a result, this yeast strain can be utilized for integrating fixed copies of genes in one transformation. They successfully demonstrated the utility of these strains by comparing 10 different norcoclaurine synthases (NCSs), each of which had one, two, three, or four copies, resulting in 40 strains being generated and evaluated in a relatively short time. Based on these strains, they unambiguously determined the best NCS and the optimum copy number to produce the highest titer of (S)-norcoclaurine (Bourgeois et al., 2018).

Recently, Baek et al. also characterized synthetic sequences (consisting of a 20-nt target sequence and 3-bp PAM sequence) and genomic loci, albeit on a smaller scale (two synthetic sequences and 12 intergenic loci) than (Bourgeois et al., 2018; Baek et al., 2021). The authors integrated the most efficient synthetic sequence into one to six intergenic loci with the highest integration efficiency, resulting in six different strains with one to six copies of the synthetic sequence in the genome (Figure 5B). To simplify the cloning process for the donor DNA, six corresponding plasmids were generated. Using the strain containing six synthetic targets, this platform successfully integrated six genes for kauniolide biosynthesis (~15 kb) into six different loci with 40% efficiency (Baek et al., 2021). So far, this is the highest number of unique gene cassettes to be integrated into unique loci in yeast (Table 1). Although Shi et al. managed to remarkably edit 18 loci of δ sites, each locus contained the same cassette (Shi et al., 2016). Similarly, Horwitz et al. astoundingly integrated 11 gene cassettes, but these cassettes were integrated into three different loci (Horwitz et al., 2015).

In another study, Qi et al. utilized the highly efficient genomic loci from Apel et al. and built a yeast strain containing eight well-defined cassettes in eight different loci (Apel et al., 2017; Qi et al., 2021). Each cassette contained a unique set of promoters and terminators with a synthetic linker in the middle, resulting in a strain with eight promoter-linker-terminator cassettes in the genome (Figure 5C). Since each linker had different sequences, unique gRNA cassettes were required to target each site, much like using multiple gRNA cassettes to target multiple sites. However, because the Cas9 cassette, promoters, and terminators were pre-integrated into the genome, the transformation only required plasmid(s) with gRNA cassettes and donor DNA genes (without promoters and terminators). This platform successfully integrated five genes in the astaxanthin biosynthetic pathway into five different loci with 69% efficiency (Qi et al., 2021).

The utilization of pre-defined endogenous or synthetic target sequences has dramatically increased the maximum number of genes that can be integrated into unique loci. This is largely because the total amount of DNA transformed into yeast can be reduced due to either shorter gRNA (typically only one is required) plasmids (Finnigan and Thorner, 2016; Shi et al., 2016; Bourgeois et al., 2018; Baek et al., 2021) or shorter donor DNAs (Qi et al., 2021). Nevertheless, this approach has two major limitations. First, the targets are pre-determined and therefore cannot be used to disrupt endogenous competing pathways. Second, constructing the base strain requires additional effort for the characterization and integration of the pre-defined synthetic sequences.

Another study that does not completely fit into these three multiplex categories but contains interesting ideas was carried out by Ferreira et al. They attempted to use off-target effects to knock-out multiple genes simultaneously (Ferreira et al., 2017). The authors built a bioinformatics tool to predict the promiscuity of gRNA sequences and used promiscuous gRNA to target multiple genes at once. Using one promiscuous gRNA, the double knockout of *FAA1* and *FAA4* was achieved with 100% efficiency (Ferreira et al., 2017). Despite this ingenious approach, there is a major drawback in the limited availability of promiscuous sequences. As one may expect, most of the promiscuous gRNA sequences are within the same gene families, transposons, or paralogs.

## Multiplex Genome Editing Using Cas12a

Cas9 is the first and most popular Cas protein for genome-editing purposes in yeast (Jinek et al., 2012; Malcı et al., 2020). However, another Cas protein, called Cas12a (previously Cpf1), has started to gain attentions for genome editing, including multiplex gene editing in yeast. There are three important differences between Cas9 and Cas12a: (i) Cas12a recognizes a 5'-(T)TTV-3' PAM sequence, and it cleaves the sequences downstream of the PAM instead of upstream like Cas9; (ii) Cas12a only needs crRNA to function as an endonuclease, instead of the crRNA and tracrRNA used by Cas9; (iii) Cas12a has an inherent capability to process pre-crRNA, whereas Cas9 requires host RNase activity for pre-crRNA processing. As shown by Bao et al. for multiplex CRISPR/Cas9 (see above), pre-crRNA processing can be useful for expressing multiple gRNAs in one cassette (Bao et al., 2015). Therefore, Cas12a has huge potential to be used for an efficient multiplex genome-editing system. For a more comprehensive comparison between Cas9 and Cas12a, the readers can refer to a recent review (Paul and Montoya, 2020).

The first utilization of Cas12a for multiplex genome editing was demonstrated in mammalian cells and the mouse brain (Zetsche et al., 2017). In yeast, at least two studies have demonstrated the utilization of Cas12a for multiplex genome integration. The first study used one plasmid containing a Cas12a cassette and crRNA array. They successfully integrated three genes (~9 kb) for the β-carotene biosynthesis pathway into three different loci with 91% efficiency (Verwaal et al., 2018). Similarly, the second study also used a one plasmid system and resulted in the integration of four genes (~13 kb) for β-carotene production into three different loci (two genes in the same locus) with up to 32% efficiency. They also integrated four genes (~7.5 kb) for the biosynthesis of the sesquiterpene, patchoulol, into three different loci (*FPPS* and *PTS* were linked) with 30% efficiency (Li et al., 2018). Although studies for multiplex genome editing using Cas12a are still rare, increasing understanding of Cas12a mechanism will accelerate the applications of this system for multiplex genome editing in yeast.

## Other Applications of Multiplex CRISPR/Cas9

Other than gene integration, multiplex CRISPR/Cas9 can also be used to optimize yeast strains *via* metabolic engineering strategies, such as gene disruption for eliminating competing pathways, gene downregulation for diminishing competing but important pathways, and gene upregulation for boosting endogenous yeast pathways (Siddiqui et al., 2012; Sander and Joung, 2014; Pyne et al., 2019). As mentioned above, multiple gene disruptions or gene deletions are usually implemented using RNA-cleaving mechanisms (HDV, tRNA, Csy4, and HI-CRISPR) due to their simplicity and high efficiencies in disrupting multiple genes (Ryan et al., 2014; Bao et al., 2015; Ferreira et al., 2018; Zhang et al., 2019). The systems for endogenous gene downregulation and upregulation are less developed, but some exciting progress has been demonstrated. CRISPR/Cas9-based gene downregulation (CRISPR interference - CRISPRi) usually exploits deactivated Cas9 (dCas9), which was generated by mutating the nucleases domains of Cas9 (Qi et al., 2013). dCas9 does not create DSBs in the target sites, but still tightly binds to the target sites, this causes repression of gene expression downstream of the target sites. This repression, which efficiency can be increased by fusion of dCas9 with different repressive chromatin modifier domains, is caused by the dCas9 sterically hindering RNA polymerase binding (Gilbert et al., 2013; Qi et al., 2013). Multiplex CRISPRi in yeast has been successfully demonstrated by simultaneously repressing seven yeast genes to increase β-amyrin production (Ni et al., 2019). The septuple site targeting efficiency was 40%, with the repression of each gene being between 60 and 80% (Ni et al., 2019). CRISPR/Cas9-based gene upregulation (CRISPR activation – CRISPRa) has also been developed by fusion of the dCas9 protein with strong transcriptional activator domains, such as VP64, p65AD, Rta, or a combination of them (Farzadfard et al., 2013). Two studies have demonstrated at least simultaneous double-gene activation using either VP64 (Zalatan et al., 2015) or V64-p65AD-Rta (Deaner et al., 2017) as the activator domains. Moreover, both studies did not only show the double activations but also show interference of other genes, which allows simultaneous activation and repression in one transformation (Zalatan et al., 2015; Deaner et al., 2017). Another study combined CRISPRa, CRISPRi, and gene deletion using optimized versions of dCas12a, dCas9, and Cas9, respectively, which they called CRISPR-AID (Lian et al., 2017). CRISPR-AID integrated the three nucleases into the genome and exploited the Csy4 system to process the gRNAs. Using this approach, upregulation of *HMG1*, downregulation of *ERG9*, and deletion of *ROX1* were achieved, and the β-carotene titer increased by 3-fold (Lian et al., 2017). More complete reviews of CRISPRi and CRISPRa are available elsewhere (Jensen, 2018).

## Perspectives

The advancement of CRISPR/Cas9 from its discovery as a bacterial immune system to multiplex genome-editing applications has been revolutionary. Despite these remarkable innovations, CRISPR/Cas9 multiplex gene integration has several challenges that need to be addressed to improve the capacity and efficiency of this technique. First, the gRNA design and target loci selection steps need to be minimized. Designing gRNAs is a crucial step for successful CRISPR/Cas9 gene editing, including multiplex gene integration (DiCarlo et al., 2013; Adiego-Pérez et al., 2019). Although the development of various assisting tools for gRNA designs has been greatly improved for various Cas proteins (Concordet and Haeussler, 2018; Labuhn et al., 2018; Labun et al., 2019; Liao et al., 2019), the *in vivo* efficiency of these gRNAs still has to be scrutinized, and this is even more significant for multiplex gene integration (Bourgeois et al., 2018). Moreover, the selection of target loci also plays a significant role in the integration efficiency. For example, Baek et al. found that certain target sites in gene-sparse loci were highly inefficient, which may be due to limited chromatin accessibility (Baek et al., 2021). Similar observations were also demonstrated elsewhere (Mans et al., 2015; Bourgeois et al., 2018; Verkuijl and Rots, 2019). The characterization of efficient gRNAs and target loci, as well as the installation of synthetic landing pads, would greatly increase the capability and efficiency of multiplex gene integration (Apel et al., 2017; Bourgeois et al., 2018; Baek et al., 2021). Second, HDR as the DSB repair mechanism in yeast needs to be optimized. Baker’s yeast is known to prefer HDR over non-homologous end joining (NHEJ) for repairing DSBs. This is demonstrated by expressing Cas9 and gRNA without donor DNA in a cell which can cause toxicity in yeast. However, some studies of multiplex gene integration showed that some yeast colonies can still survive without donor DNAs, indicating that NHEJ operates to repair the DSBs (Baek et al., 2021). This can cause an increase in false positive colonies and reduce the efficiency of multiplex genes integration. Deletions of some genes involved in the NHEJ pathway, such as *POL4*, *DNL4*, and *Ku70*, were shown to reduce false positive rates and increase the success of HDR (Lemos et al., 2018; Yan and Finnigan, 2018). Therefore, the deletion of the NHEJ genes may increase the capability and efficiency of multiplex genome integration by reducing the number of background colonies, generated by chromosome repairs by NHEJ. Third, yeast transformation efficiency needs to be improved. CRISPR/Cas9 multiplex gene integration in yeast requires the introduction of a large amount of foreign DNA (donor DNA cassettes, gRNA plasmids, and Cas9 plasmids) to yeast cells. The more targets to be edited, the more donor DNA and/or gRNA cassettes will be required. Transformation techniques, such as electroporation or the addition of amino acids, can be incorporated to improve the transformation efficiency (Benatuil et al., 2010; Yu et al., 2019). Finally, the development of CRISPR/Cas9 multiplex genome integration in yeast can be improved by combining multiple methods. For example, the combination of the pre-installed target sites (Bourgeois et al., 2018; Baek et al., 2021) with RNA cleaving mechanisms (Bao et al., 2015; Shi et al., 2016; Ferreira et al., 2018) or with a sequential integration approach (Li et al., 2020) can minimize the required time and maximize the number of genes for multiplex integration. The currently developed methods (Table 1) have been remarkable and have endless potential. However, the success of addressing these limitations and creatively combining several methods will expand the scope of multiplex genome editing. In turn, this will accelerate the study and optimization of complex specialized metabolic pathways in yeast.

## Author Contributions

JCU and D-KR conceived the work. JCU and CH wrote the manuscript. JCU prepared the figures. D-KR revised the manuscript. All the authors read and approved the final version of the manuscript.

## Conflict of Interest

The authors declare that the research was conducted in the absence of any commercial or financial relationships that could be construed as a potential conflict of interest.

## Publisher’s Note

All claims expressed in this article are solely those of the authors and do not necessarily represent those of their affiliated organizations, or those of the publisher, the editors and the reviewers. Any product that may be evaluated in this article, or claim that may be made by its manufacturer, is not guaranteed or endorsed by the publisher.

## Footnotes

^1^ http://yeastriction.tnw.tudelft.nl/

^2^ http://staff.biosustain.dtu.dk/laeb/crispy/

## References

[ref1] Adiego-Pérez B. Randazzo P. Daran J. M. Verwaal R. Roubos J. A. Daran-Lapujade P.. (2019). Multiplex genome editing of microorganisms using CRISPR-Cas. FEMS Microbiol. Lett. 366:fnz086. 10.1093/femsle/fnz086, PMID: 31087001 PMC6522427

[ref2] Apel A. R. d'Espaux L. Wehrs M. Sachs D. Li R. A. Tong G. J.. (2017). A Cas9-based toolkit to program gene expression in *Saccharomyces cerevisiae*. Nucleic Acids Res. 45, 496–508. 10.1093/nar/gkw1023, PMID: 27899650 PMC5224472

[ref3] Baek S. Utomo J. C. Lee J. Y. Dalal K. Yoon Y. J. Ro D.- K. (2021). The yeast platform engineered for synthetic gRNA-landing pads enables multiple gene integrations by a single gRNA/Cas9 system. Metab. Eng. 64, 111–121. 10.1016/j.ymben.2021.01.011, PMID: 33549837

[ref4] Bao Z. Xiao H. Liang J. Zhang L. Xiong X. Sun N.. (2015). Homology-integrated CRISPR–Cas (HI-CRISPR) system for one-step multigene disruption in *Saccharomyces cerevisiae*. ACS Synth. Biol. 4, 585–594. 10.1021/sb500255k, PMID: 25207793

[ref5] Barrangou R. Fremaux C. Deveau H. Richards M. Boyaval P. Moineau S.. (2007). CRISPR provides acquired resistance against viruses in prokaryotes. Science 315, 1709–1712. 10.1126/science.1138140, PMID: 17379808

[ref6] Bathe U. Tissier A. (2019). Cytochrome P450 enzymes: a driving force of plant diterpene diversity. Phytochemistry 161, 149–162. 10.1016/j.phytochem.2018.12.003, PMID: 30733060

[ref7] Benatuil L. Perez J. M. Belk J. Hsieh C.- M. (2010). An improved yeast transformation method for the generation of very large human antibody libraries. Protein Eng. Des. Sel. 23, 155–159. 10.1093/protein/gzq002, PMID: 20130105

[ref8] Bond C. M. Tang Y. (2019). Engineering *Saccharomyces cerevisiae* for production of simvastatin. Metab. Eng. 51, 1–8. 10.1016/j.ymben.2018.09.005, PMID: 30213650 PMC6348118

[ref9] Bourgeois L. Pyne M. E. Martin V. J. J. (2018). A highly characterized synthetic landing pad system for precise multicopy gene integration in yeast. ACS Synth. Biol. 7, 2675–2685. 10.1021/acssynbio.8b00339, PMID: 30372609

[ref10] Chao R. Yuan Y. Zhao H. (2015). Recent advances in DNA assembly technologies. FEMS Yeast Res. 15, 1–9. 10.1111/1567-1364.12171, PMID: 24903193 PMC4257898

[ref11] Chapple C. (1998). Molecular-genetic analysis of plant cytochrome P450-dependent monooxygenases. Annu. Rev. Plant Physiol. Plant Mol. Biol. 49, 311–343. 10.1146/annurev.arplant.49.1.311, PMID: 15012237

[ref12] Concordet J.-P. Haeussler M. (2018). CRISPOR: intuitive guide selection for CRISPR/Cas9 genome editing experiments and screens. Nucleic Acids Res. 46, W242–W245. 10.1093/nar/gky354, PMID: 29762716 PMC6030908

[ref13] Cong L. Ran F. A. Cox D. Lin S. Barretto R. Habib N.. (2013). Multiplex genome engineering using CRISPR/Cas systems. Science 339, 819–823. 10.1126/science.1231143, PMID: 23287718 PMC3795411

[ref14] Da Silva N. A. Srikrishnan S. (2012). Introduction and expression of genes for metabolic engineering applications in *Saccharomyces cerevisiae*. FEMS Yeast Res. 12, 197–214. 10.1111/j.1567-1364.2011.00769.x, PMID: 22129153

[ref15] David F. Siewers V. (2015). Advances in yeast genome engineering. FEMS Yeast Res. 15, 1–14. 10.1111/1567-1364.12200, PMID: 25154295

[ref16] Deaner M. Alper H. S. (2019). Enhanced scale and scope of genome engineering and regulation using CRISPR/Cas in *Saccharomyces cerevisiae*. FEMS Yeast Res. 19:foz076. 10.1093/femsyr/foz076, PMID: 31665284

[ref17] Deaner M. Mejia J. Alper H. S. (2017). Enabling graded and large-scale multiplex of desired genes using a dual-mode dCas9 activator in *Saccharomyces cerevisiae*. ACS Synth. Biol. 6, 1931–1943. 10.1021/acssynbio.7b00163, PMID: 28700213

[ref18] Deltcheva E. Chylinski K. Sharma C. M. Gonzales K. Chao Y. Pirzada Z. A.. (2011). CRISPR RNA maturation by trans-encoded small RNA and host factor RNase III. Nature 471, 602–607. 10.1038/nature09886, PMID: 21455174 PMC3070239

[ref19] DiCarlo J. E. Norville J. E. Mali P. Rios X. Aach J. Church G. M. (2013). Genome engineering in *Saccharomyces cerevisiae* using CRISPR-Cas systems. Nucleic Acids Res. 41, 4336–4343. 10.1093/nar/gkt135, PMID: 23460208 PMC3627607

[ref20] Doudna J. A. Charpentier E. (2014). The new frontier of genome engineering with CRISPR-Cas9. Science 346:1258096. 10.1126/science.1258096, PMID: 25430774

[ref21] Farzadfard F. Perli S. D. Lu T. K. (2013). Tunable and multifunctional eukaryotic transcription factors based on CRISPR/Cas. ACS Synth. Biol. 2, 604–613. 10.1021/sb400081r, PMID: 23977949 PMC3805333

[ref22] Ferreira R. Gatto F. Nielsen J. (2017). Exploiting off-targeting in guide-RNAs for CRISPR systems for simultaneous editing of multiple genes. FEBS Lett. 591, 3288–3295. 10.1002/1873-3468.12835, PMID: 28884816

[ref23] Ferreira R. Skrekas C. Nielsen J. David F. (2018). Multiplexed CRISPR/Cas9 genome editing and gene regulation using Csy4 in *Saccharomyces cerevisiae*. ACS Synth. Biol. 7, 10–15. 10.1021/acssynbio.7b00259, PMID: 29161506

[ref24] Finnigan G. C. Thorner J. (2016). mCAL: a new approach for versatile multiplex action of Cas9 using one sgRNA and loci flanked by a programmed target sequence. G3 6, 2147–2156. 10.1534/g3.116.029801, PMID: 27185399 PMC4938667

[ref25] Gardner J. M. Jaspersen S. L. (2014). “Manipulating the yeast genome: deletion, mutation, and tagging by PCR,” in Yeast Genetics: Methods and Protocols. eds. Smith J. S. Burke D. J. (New York, NY: Springer), 45–78. 10.1007/978-1-4939-1363-3_5 25213239

[ref26] Gilbert L. A. Larson M. H. Morsut L. Liu Z. Brar G. A. Torres S. E.. (2013). CRISPR-mediated modular RNA-guided regulation of transcription in eukaryotes. Cell 154, 442–451. 10.1016/j.cell.2013.06.044, PMID: 23849981 PMC3770145

[ref27] Hafner J. Payne J. Mohammadi P. H. Hatzimanikatis V. Smolke C. (2021). A computational workflow for the expansion of heterologous biosynthetic pathways to natural product derivatives. Nat. Commun. 12:1760. 10.1038/s41467-021-22022-5, PMID: 33741955 PMC7979880

[ref28] Hanasaki M. Masumoto H. (2019). CRISPR/transposon gene integration (CRITGI) can manage gene expression in a retrotransposon-dependent manner. Sci. Rep. 9:15300. 10.1038/s41598-019-51891-6, PMID: 31653950 PMC6814769

[ref29] Haurwitz R. E. Jinek M. Wiedenheft B. Zhou K. Doudna J. A. (2010). Sequence- and structure-specific RNA processing by a CRISPR endonuclease. Science 329, 1355–1358. 10.1126/science.1192272, PMID: 20829488 PMC3133607

[ref30] Horwitz A. A. Walter J. M. Schubert M. G. Kung S. H. Hawkins K. Platt D. M.. (2015). Efficient multiplexed integration of synergistic alleles and metabolic pathways in yeasts *via* CRISPR-Cas. Cell Syst. 1, 88–96. 10.1016/j.cels.2015.02.001, PMID: 27135688

[ref31] Hou S. Qin Q. Dai J. (2018). Wicket: a versatile tool for the integration and optimization of exogenous pathways in *Saccharomyces cerevisiae*. ACS Synth. Biol. 7, 782–788. 10.1021/acssynbio.7b00391, PMID: 29474063

[ref32] Huang S. Geng A. (2020). High-copy genome integration of 2,3-butanediol biosynthesis pathway in *Saccharomyces cerevisiae via in vivo* DNA assembly and replicative CRISPR-Cas9 mediated delta integration. J. Biotechnol. 310, 13–20. 10.1016/j.jbiotec.2020.01.014, PMID: 32006629

[ref33] Ishino Y. Shinagawa H. Makino K. Amemura M. Nakata A. (1987). Nucleotide sequence of the *iap* gene, responsible for alkaline phosphatase isozyme conversion in *Escherichia coli*, and identification of the gene product. J. Bacteriol. 169, 5429–5433. 10.1128/jb.169.12.5429-5433.1987, PMID: 3316184 PMC213968

[ref34] Jakočiūnas T. Bonde I. Herrgård M. Harrison S. J. Kristensen M. Pedersen L. E.. (2015b). Multiplex metabolic pathway engineering using CRISPR/Cas9 in *Saccharomyces cerevisiae*. Metab. Eng. 28, 213–222. 10.1016/j.ymben.2015.01.008, PMID: 25638686

[ref35] Jakočiūnas T. Jensen M. K. Keasling J. D. (2016). CRISPR/Cas9 advances engineering of microbial cell factories. Metab. Eng. 34, 44–59. 10.1016/j.ymben.2015.12.003, PMID: 26707540

[ref36] Jakočiūnas T. Rajkumar A. S. Zhang J. Arsovska D. Rodriguez A. Jendresen C. B.. (2015a). CasEMBLR: Cas9-facilitated multiloci genomic integration of *in vivo* assembled DNA parts in *Saccharomyces cerevisiae*. ACS Synth. Biol. 4, 1226–1234. 10.1021/acssynbio.5b00007, PMID: 25781611

[ref37] Jansen R. Embden J. D. A. Gaastra W. Schouls L. M. (2002). Identification of genes that are associated with DNA repeats in prokaryotes. Mol. Microbiol. 43, 1565–1575. 10.1046/j.1365-2958.2002.02839.x, PMID: 11952905

[ref38] Jensen M. K. (2018). Design principles for nuclease-deficient CRISPR-based transcriptional regulators. FEMS Yeast Res. 18:foy039. 10.1093/femsyr/foy039, PMID: 29726937 PMC5932555

[ref39] Jensen N. B. Strucko T. Kildegaard K. R. David F. Maury J. Mortensen U. H.. (2014). EasyClone: method for iterative chromosomal integration of multiple genes *Saccharomyces cerevisiae*. FEMS Yeast Res. 14, 238–248. 10.1111/1567-1364.12118, PMID: 24151867 PMC4282123

[ref40] Jessop-Fabre M. M. Jakočiūnas T. Stovicek V. Dai Z. Jensen M. K. Keasling J. D.. (2016). EasyClone-markerfree: a vector toolkit for marker-less integration of genes into *Saccharomyces cerevisiae via* CRISPR-Cas9. Biotechnol. J. 11, 1110–1117. 10.1002/biot.201600147, PMID: 27166612 PMC5094547

[ref41] Jinek M. Chylinski K. Fonfara I. Hauer M. Doudna J. A. Charpentier E. (2012). A programmable dual-RNA–guided DNA endonuclease in adaptive bacterial immunity. Science 337, 816–821. 10.1126/science.1225829, PMID: 22745249 PMC6286148

[ref42] Jinek M. Jiang F. Taylor D. W. Sternberg S. H. Kaya E. Ma E.. (2014). Structures of Cas9 endonucleases reveal RNA-mediated conformational activation. Science 343:1247997. 10.1126/science.1247997, PMID: 24505130 PMC4184034

[ref43] Kotopka B. J. Smolke C. D. (2019). Production of the cyanogenic glycoside dhurrin in yeast. Metab. Eng. Commun. 9:e00092. 10.1016/j.mec.2019.e00092, PMID: 31110942 PMC6512747

[ref44] Krastanova O. Hadzhitodorov M. Pesheva M. (2005). Ty elements of the yeast *Saccharomyces cerevisiae*. Biotechnol. Biotechnol. Equip. 19, 19–26. 10.1080/13102818.2005.10817272

[ref45] Labuhn M. Adams F. F. Ng M. Knoess S. Schambach A. Charpentier E. M.. (2018). Refined sgRNA efficacy prediction improves large- and small-scale CRISPR–Cas9 applications. Nucleic Acids Res. 46, 1375–1385. 10.1093/nar/gkx1268, PMID: 29267886 PMC5814880

[ref46] Labun K. Montague T. G. Krause M. Torres C. Y. N. Tjeldnes H. Valen E. (2019). CHOPCHOP v3: expanding the CRISPR web toolbox beyond genome editing. Nucleic Acids Res. 47, W171–W174. 10.1093/nar/gkz365, PMID: 31106371 PMC6602426

[ref47] Lee M. E. DeLoache W. C. Cervantes B. Dueber J. E. (2015). A highly characterized yeast toolkit for modular, multipart assembly. ACS Synth. Biol. 4, 975–986. 10.1021/sb500366v, PMID: 25871405

[ref48] Lemos B. R. Kaplan A. C. Bae J. E. Ferrazzoli A. E. Kuo J. Anand R. P.. (2018). CRISPR/Cas9 cleavages in budding yeast reveal templated insertions and strand-specific insertion/deletion profiles. Proc. Natl. Acad. Sci. U. S. A. 115, E2040–E2047. 10.1073/pnas.1716855115, PMID: 29440496 PMC5834694

[ref49] Leonard E. Koffas M. A. G. (2007). Engineering of artificial plant cytochrome P450 enzymes for synthesis of isoflavones by *Escherichia coli*. Appl. Environ. Microbiol. 73, 7246–7251. 10.1128/AEM.01411-07, PMID: 17905887 PMC2168208

[ref50] Li Z.- H. Meng H. Ma B. Tao X. Liu M. Wang F.- Q.. (2020). Immediate, multiplexed and sequential genome engineering facilitated by CRISPR/Cas9 in *Saccharomyces cerevisiae*. J. Ind. Microbiol. Biotechnol. 47, 83–96. 10.1007/s10295-019-02251-w, PMID: 31768773

[ref51] Li Z.- H. Wang F.- Q. Wei D.- Z. (2018). Self-cloning CRISPR/Cpf1 facilitated genome editing in *Saccharomyces cerevisiae*. Bioresour. Bioprocess. 5:36. 10.1186/s40643-018-0222-8

[ref52] Lian J. HamediRad M. Hu S. Zhao H. (2017). Combinatorial metabolic engineering using an orthogonal tri-functional CRISPR system. Nat. Commun. 8:1688. 10.1038/s41467-017-01695-x, PMID: 29167442 PMC5700065

[ref53] Liao C. Ttofali F. Slotkowski R. A. Denny S. R. Cecil T. D. Leenay R. T.. (2019). Modular one-pot assembly of CRISPR arrays enables library generation and reveals factors influencing crRNA biogenesis. Nat. Commun. 10:2948. 10.1038/s41467-019-10747-3, PMID: 31270316 PMC6610086

[ref54] Makarova K. S. Wolf Y. I. Iranzo J. Shmakov S. A. Alkhnbashi O. S. Brouns S. J. J.. (2020). Evolutionary classification of CRISPR–Cas systems: a burst of class 2 and derived variants. Nat. Rev. Microbiol. 18, 67–83. 10.1038/s41579-019-0299-x, PMID: 31857715 PMC8905525

[ref55] Malcı K. Walls L. E. Rios-Solis L. (2020). Multiplex genome engineering methods for yeast cell factory development. Front. Bioeng. Biotechnol. 8:589468. 10.3389/fbioe.2020.589468, PMID: 33195154 PMC7658401

[ref56] Mali P. Yang L. Esvelt K. M. Aach J. Guell M. DiCarlo J. E.. (2013). RNA-guided human genome engineering *via* Cas9. Science 339, 823–826. 10.1126/science.1232033, PMID: 23287722 PMC3712628

[ref57] Mans R. van Rossum H. M. Wijsman M. Backx A. Kuijpers N. G. A. van den Broek M.. (2015). CRISPR/Cas9: a molecular Swiss army knife for simultaneous introduction of multiple genetic modifications in *Saccharomyces cerevisiae*. FEMS Yeast Res. 15:fov004. 10.1093/femsyr/fov004, PMID: 25743786 PMC4399441

[ref58] McCarty N. S. Shaw W. M. Ellis T. Ledesma-Amaro R. (2019). Rapid assembly of gRNA arrays *via* modular cloning in yeast. ACS Synth. Biol. 8, 906–910. 10.1021/acssynbio.9b00041, PMID: 30939239

[ref59] Meng J. Qiu Y. Shi S. (2020). CRISPR/Cas9 systems for the development of *Saccharomyces cerevisiae* cell factories. Front. Bioeng. Biotechnol. 8:594347. 10.3389/fbioe.2020.594347, PMID: 33330425 PMC7710542

[ref60] Mojica F. J. M. Díez-Villaseñor C. García-Martínez J. Soria E. (2005). Intervening sequences of regularly spaced prokaryotic repeats derive from foreign genetic elements. J. Mol. Evol. 60, 174–182. 10.1007/s00239-004-0046-3, PMID: 15791728

[ref61] Ni J. Zhang G. Qin L. Li J. Li C. (2019). Simultaneously down-regulation of multiplex branch pathways using CRISPRi and fermentation optimization for enhancing β-amyrin production in *Saccharomyces cerevisiae*. Synth. Syst. Biotechnol. 4, 79–85. 10.1016/j.synbio.2019.02.002, PMID: 30949594 PMC6428687

[ref62] Park S.- H. Hahn J.- S. (2019). Development of an efficient cytosolic isobutanol production pathway in *Saccharomyces cerevisiae* by optimizing copy numbers and expression of the pathway genes based on the toxic effect of α-acetolactate. Sci. Rep. 9:3996. 10.1038/s41598-019-40631-5, PMID: 30850698 PMC6408573

[ref63] Paul B. Montoya G. (2020). CRISPR-Cas12a: functional overview and applications. Biom. J. 43, 8–17. 10.1016/j.bj.2019.10.005, PMID: 32200959 PMC7090318

[ref64] Pyne M. E. Narcross L. Martin V. J. J. (2019). Engineering plant secondary metabolism in microbial systems. Plant Physiol. 179, 844–861. 10.1104/pp.18.01291, PMID: 30643013 PMC6393802

[ref65] Qi L. Haurwitz R. E. Shao W. Doudna J. A. Arkin A. P. (2012). RNA processing enables predictable programming of gene expression. Nat. Biotechnol. 30, 1002–1006. 10.1038/nbt.2355, PMID: 22983090

[ref66] Qi L. S. Larson M. H. Gilbert L. A. Doudna J. A. Weissman J. S. Arkin A. P.. (2013). Repurposing CRISPR as an RNA-guided platform for sequence-specific control of gene expression. Cell 152, 1173–1183. 10.1016/j.cell.2013.02.022, PMID: 23452860 PMC3664290

[ref67] Qi M. Zhang B. Jiang L. Xu S. Dong C. Du Y.- L.. (2021). PCR & go: a pre-installed expression chassis for facile integration of multi-gene biosynthetic pathways. Front. Bioeng. Biotechnol. 8:613771. 10.3389/fbioe.2020.613771, PMID: 33520963 PMC7841387

[ref68] Ronda C. Maury J. Jakočiūnas T. Baallal J. S. A. Germann S. M. Harrison S. J.. (2015). CrEdit: CRISPR mediated multi-loci gene integration in *Saccharomyces cerevisiae*. Microb. Cell Factories 14:97. 10.1186/s12934-015-0288-3, PMID: 26148499 PMC4492099

[ref69] Ryan O. W. Skerker J. M. Maurer M. J. Li X. Tsai J. C. Poddar S.. (2014). Selection of chromosomal DNA libraries using a multiplex CRISPR system. elife 3:e03703. 10.7554/eLife.03703, PMID: 25139909 PMC4161972

[ref70] Sander J. D. Joung J. K. (2014). CRISPR-Cas systems for editing, regulating and targeting genomes. Nat. Biotechnol. 32, 347–355. 10.1038/nbt.2842, PMID: 24584096 PMC4022601

[ref71] Sapranauskas R. Gasiunas G. Fremaux C. Barrangou R. Horvath P. Siksnys V. (2011). The *Streptococcus thermophilus* CRISPR/Cas system provides immunity in *Escherichia coli*. Nucleic Acids Res. 39, 9275–9282. 10.1093/nar/gkr606, PMID: 21813460 PMC3241640

[ref72] Shao Z. Zhao H. (2009). DNA assembler, an *in vivo* genetic method for rapid construction of biochemical pathways. Nucleic Acids Res. 37:e16. 10.1093/nar/gkn991, PMID: 19074487 PMC2632897

[ref73] Shi S. Liang Y. Zhang M. M. Ang E. L. Zhao H. (2016). A highly efficient single-step, markerless strategy for multi-copy chromosomal integration of large biochemical pathways in *Saccharomyces cerevisiae*. Metab. Eng. 33, 19–27. 10.1016/j.ymben.2015.10.011, PMID: 26546089

[ref74] Si T. Chao R. Min Y. Wu Y. Ren W. Zhao H. (2017). Automated multiplex genome-scale engineering in yeast. Nat. Commun. 8:15187. 10.1038/ncomms15187, PMID: 28469255 PMC5418614

[ref75] Siddiqui M. S. Thodey K. Trenchard I. Smolke C. D. (2012). Advancing secondary metabolite biosynthesis in yeast with synthetic biology tools. FEMS Yeast Res. 12, 144–170. 10.1111/j.1567-1364.2011.00774.x, PMID: 22136110

[ref76] Srinivasan P. Smolke C. D. (2019). Engineering a microbial biosynthesis platform for *de novo* production of tropane alkaloids. Nat. Commun. 10:3634. 10.1038/s41467-019-11588-w, PMID: 31406117 PMC6690885

[ref77] Srinivasan P. Smolke C. D. (2020). Biosynthesis of medicinal tropane alkaloids in yeast. Nature 585, 614–619. 10.1038/s41586-020-2650-9, PMID: 32879484 PMC7529995

[ref78] Storici F. Durham C. L. Gordenin D. A. Resnick M. A. (2003). Chromosomal site-specific double-strand breaks are efficiently targeted for repair by oligonucleotides in yeast. Proc. Natl. Acad. Sci. U. S. A. 100, 14994–14999. 10.1073/pnas.2036296100, PMID: 14630945 PMC299876

[ref79] Stovicek V. Holkenbrink C. Borodina I. (2017). CRISPR/Cas system for yeast genome engineering: advances and applications. FEMS Yeast Res. 17:fox030. 10.1093/femsyr/fox030, PMID: 28505256 PMC5812514

[ref80] Tang T.- H. Bachellerie J.- P. Rozhdestvensky T. Bortolin M.- L. Huber H. Drungowski M.. (2002). Identification of 86 candidates for small non-messenger RNAs from the archaeon *Archaeoglobus fulgidus*. Proc. Natl. Acad. Sci. U. S. A. 99, 7536–7541. 10.1073/pnas.112047299, PMID: 12032318 PMC124276

[ref81] Venema J. Tollervey D. (1999). Ribosome synthesis in *Saccharomyces cerevisiae*. Annu. Rev. Genet. 33, 261–311. 10.1146/annurev.genet.33.1.261, PMID: 10690410

[ref82] Verkuijl S. A. N. Rots M. G. (2019). The influence of eukaryotic chromatin state on CRISPR–Cas9 editing efficiencies. Curr. Opin. Biotechnol. 55, 68–73. 10.1016/j.copbio.2018.07.005, PMID: 30189348

[ref83] Verwaal R. Buiting-Wiessenhaan N. Dalhuijsen S. Roubos J. A. (2018). CRISPR/Cpf1 enables fast and simple genome editing of *Saccharomyces cerevisiae*. Yeast 35, 201–211. 10.1002/yea.3278, PMID: 28886218 PMC5836994

[ref84] Walter J. M. Chandran S. S. Horwitz A. A. (2016). CRISPR-Cas-assisted multiplexing (CAM): simple same-day multi-locus engineering in yeast. J. Cell. Physiol. 231, 2563–2569. 10.1002/jcp.25375, PMID: 26991244

[ref85] Wang L. Deng A. Zhang Y. Liu S. Liang Y. Bai H.. (2018). Efficient CRISPR–Cas9 mediated multiplex genome editing in yeasts. Biotechnol. Biofuels 11:277. 10.1186/s13068-018-1271-0, PMID: 30337956 PMC6180501

[ref86] Xie K. Minkenberg B. Yang Y. (2015). Boosting CRISPR/Cas9 multiplex editing capability with the endogenous tRNA-processing system. Proc. Natl. Acad. Sci. U. S. A. 112, 3570–3575. 10.1073/pnas.1420294112, PMID: 25733849 PMC4371917

[ref87] Yan Y. Finnigan G. C. (2018). Development of a multi-locus CRISPR gene drive system in budding yeast. Sci. Rep. 8:17277. 10.1038/s41598-018-34909-3, PMID: 30467400 PMC6250742

[ref88] Yang Z. Blenner M. (2020). Genome editing systems across yeast species. Curr. Opin. Biotechnol. 66, 255–266. 10.1016/j.copbio.2020.08.011, PMID: 33011454 PMC7744358

[ref89] Yee D. A. DeNicola A. B. Billingsley J. M. Creso J. G. Subrahmanyam V. Tang Y. (2019). Engineered mitochondrial production of monoterpenes in *Saccharomyces cerevisiae*. Metab. Eng. 55, 76–84. 10.1016/j.ymben.2019.06.004, PMID: 31226348 PMC6717016

[ref90] Yu S.- C. Kuemmel F. Skoufou-Papoutsaki M.- N. Spanu P. D. (2019). Yeast transformation efficiency is enhanced by TORC1- and eisosome-dependent signaling. Microbiol. Open 8:e00730. 10.1002/mbo3.730, PMID: 30311441 PMC6528558

[ref91] Zalatan J. G. Lee M. E. Almeida R. Gilbert L. A. Whitehead E. H. La R. M.. (2015). Engineering complex synthetic transcriptional programs with CRISPR RNA scaffolds. Cell 160, 339–350. 10.1016/j.cell.2014.11.052, PMID: 25533786 PMC4297522

[ref92] Zetsche B. Heidenreich M. Mohanraju P. Fedorova I. Kneppers J. DeGennaro E. M.. (2017). Multiplex gene editing by CRISPR–Cpf1 using a single crRNA array. Nat. Biotechnol. 35, 31–34. 10.1038/nbt.3737, PMID: 27918548 PMC5225075

[ref93] Zhang Y. Su M. Qin N. Nielsen J. Liu Z. (2020). Expressing a cytosolic pyruvate dehydrogenase complex to increase free fatty acid production in *Saccharomyces cerevisiae*. Microb. Cell Fact. 19:226. 10.1186/s12934-020-01493-z, PMID: 33302960 PMC7730738

[ref94] Zhang Y. Wang J. Wang Z. Zhang Y. Shi S. Nielsen J.. (2019). A gRNA-tRNA array for CRISPR-Cas9 based rapid multiplexed genome editing in *Saccharomyces cerevisiae*. Nat. Commun. 10:1053. 10.1038/s41467-019-09005-3, PMID: 30837474 PMC6400946

